# Solution Structure and Dynamics of the I214V Mutant of the Rabbit Prion Protein

**DOI:** 10.1371/journal.pone.0013273

**Published:** 2010-10-07

**Authors:** Yi Wen, Jun Li, Minqian Xiong, Yu Peng, Wenming Yao, Jing Hong, Donghai Lin

**Affiliations:** 1 NMR Laboratory, Shanghai Institute of Materia Medica, Chinese Academy of Sciences, Shanghai, China; 2 The Key Laboratory of Chemical Biology of Fujian Province, College of Chemistry and Chemical Engineering, Xiamen University, Xiamen, China; Indiana University, United States of America

## Abstract

**Background:**

The conformational conversion of the host-derived cellular prion protein (PrP^C^) into the disease-associated scrapie isoform (PrP^Sc^) is responsible for the pathogenesis of transmissible spongiform encephalopathies (TSEs). Various single-point mutations in PrP^C^s could cause structural changes and thereby distinctly influence the conformational conversion. Elucidation of the differences between the wild-type rabbit PrP^C^ (RaPrP^C^) and various mutants would be of great help to understand the ability of RaPrP^C^ to be resistant to TSE agents.

**Methodology/Principal Findings:**

We determined the solution structure of the I214V mutant of RaPrP^C^(91–228) and detected the backbone dynamics of its structured C-terminal domain (121–228). The I214V mutant displays a visible shift of surface charge distribution that may have a potential effect on the binding specificity and affinity with other chaperones. The number of hydrogen bonds declines dramatically. Urea-induced transition experiments reveal an obvious decrease in the conformational stability. Furthermore, the NMR dynamics analysis discloses a significant increase in the backbone flexibility on the pico- to nanosecond time scale, indicative of lower energy barrier for structural rearrangement.

**Conclusions/Significance:**

Our results suggest that both the surface charge distribution and the intrinsic backbone flexibility greatly contribute to species barriers for the transmission of TSEs, and thereby provide valuable hints for understanding the inability of the conformational conversion for RaPrP^C^.

## Introduction

The conformational conversion of the prion protein, from the normal cellular form (PrP^C^) to the abnormal scrapie isoform (PrP^Sc^), is responsible for the pathogenesis of transmissible spongiform encephalopathies (TSEs) [1]. The “protein-only” hypothesis is supported by much compelling evidence presented recently [2]–[4]. To elucidate the detailed mechanism of the conformational change, understanding of the structural basis of the prion protein is required. The three-dimensional structures of PrP^C^ among species have been extensively determined using NMR techniques so far [5]–[16]. Great effort has also been made to explore the architecture of PrP^Sc^ or PrP^Sc^-like filaments [17]–[21].

Noticeably, all known forms of inherited human TSEs, including Creutzfeldt-Jakob disease (CJD), Gerstmann-Straussler-Scheinker syndrome (GSS) and fatal familial insomnia (FFI), are closely associated with dominant mutations in human PrP^C^ (hPrP^C^) [22], [23]. In addition to apparently disease-causing mutations in humans, polymorphisms in sheep appear to exert substantial effects on variations of PrP^C^ in disease susceptibility [24]–[27]. Particularly, rabbits are one of the few mammalian animals reported to be resistant to TSE agents [28]. Multiple amino acid residues throughout the rabbit PrP^C^ (RaPrP^C^) sequence contribute to the inability of RaPrP^C^ to form PrP^Sc^. Several mouse PrP^C^ (mPrP^C^) mutants, with one single residue substituted by the corresponding residue in RaPrP^C^, are completely inhibited to undergo the conversion to the disease-related isoform [29]. All these facts indicate a significant influence on the property of the PrP^C^ structure as the result of one important residue mutation. Therefore, detailed comparisons of three-dimensional structures of wild-type PrP^C^ and its mutants could provide valuable insights into the underlying molecular mechanism of prion conversions.

In our previous work we have demonstrated that the S173N substitution leads to distinct structural changes for RaPrP^C^ [30]. However, whether similar changes could be observed in other single-point mutants remains to be addressed. Since mPrP^C^ with the V214I substitution is prohibited to convert to the abnormal form [29], we thereby expect that the I214V substitution would cause structural changes more or less for RaPrP^C^. In this present work, we determined the solution structure of the I214V mutant of RaPrP^C^(91–228) using multi-dimensional heteronuclear NMR techniques. In addition, we performed ^15^N relaxation measurements to detect the backbone dynamics of its structured C-terminal domain (121–228). Furthermore, we investigated its structural stability using CD spectroscopy. Our results reveal significant structural changes caused by the single-residue mutation, which may be of benefit to understand the detailed molecular mechanism of the conformational conversion for prion proteins.

## Results

### Solution structure

**Figure 1:**
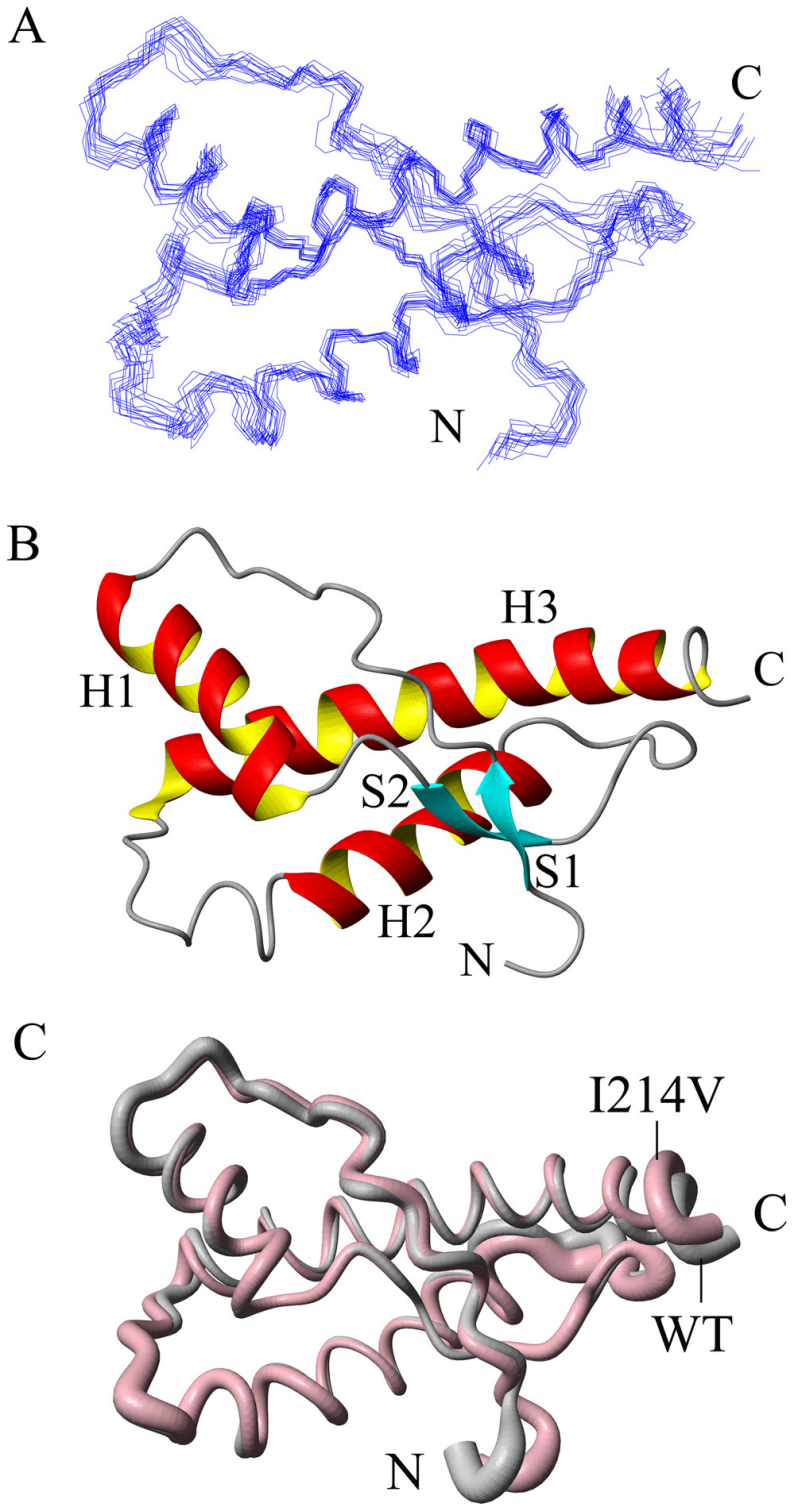
Solution structure of the I214V mutant of RaPrP^C^(91–228). (A) Diagram of 15 lowest-energy conformations. (B) Ribbon cartoon showing the secondary structure elements of the mean structure. (C) Sausage diagram showing the superposition of structures of the wild-type (grey) and the mutant (pink). The structure was determined in acetate buffer at pH 4.5. The diagrams are generated using MolMol [61].

A family of 200 structures is calculated and the structural statistics are presented in Table 1. The diagram representing 15 lowest-energy structures for the I214V mutant of RaPrP^C^(91–228) in solution is shown in Fig. 1A, together with a ribbon cartoon of average secondary structure elements displayed in Fig. 1B. The I214 mutant contains two short antiparallel β-sheets (S1: 128–130, S2: 160–162) and three α-helices (H1: 144–155, H2: 175–186, H3:199–227), with a disulfide bond (C178–C213) stabilizing helices 2 and 3. The N-terminal loop 91–120 is highly disordered. Loop 165–172 is well defined owing to the long-range NOEs from residues at the end of helix 3. The overall structure of the I214V mutant appears to be identical to that of the wild-type (Fig. 1C).

**Table 1:** Structural statistics of the I214V mutant of RaPrP^C^(91–228).

Quantity	Value
Distance restraints	
Intraresidue (| i-j | = 0)	703
Sequential (| i-j | = 1)	684
Medium range (2<| i-j |<4)	627
Long range (| i-j |>5)	717
Total	2731
Dihedral angle restraints (Φ and Ψ)^a^	134
Hydrogen bond restraints	54
Restraints violations (15 structures)	
NOE distance violation (>0.3 Å)	0
Torsion angle violation (>5°)	0
RMSD from mean structure (Å)	
All residues (backbone atoms)	0.87±0.22
Secondary structures (backbone atoms)	0.67±0.20
All residues (heavy atoms)	1.62±0.26
Secondary structures (heavy atoms)	1.43±0.24
Ramachandran analysis (%) (124–228)^b^	
Residues in most favored regions	77.9
Residues in additionally allowed regions	17.4
Residues in generously allowed regions	2.8
Residues in disallowed regions	1.9

a The dihedral angle restraints are generated from secondary structure determined by the program CSI (David Wishart, Brian Sykes, Leigh Willard, Tim Jellard and reference [64]).

b Residues included in the analysis are indicated in parentheses.

### Electrostatic potential

**Figure 2:**
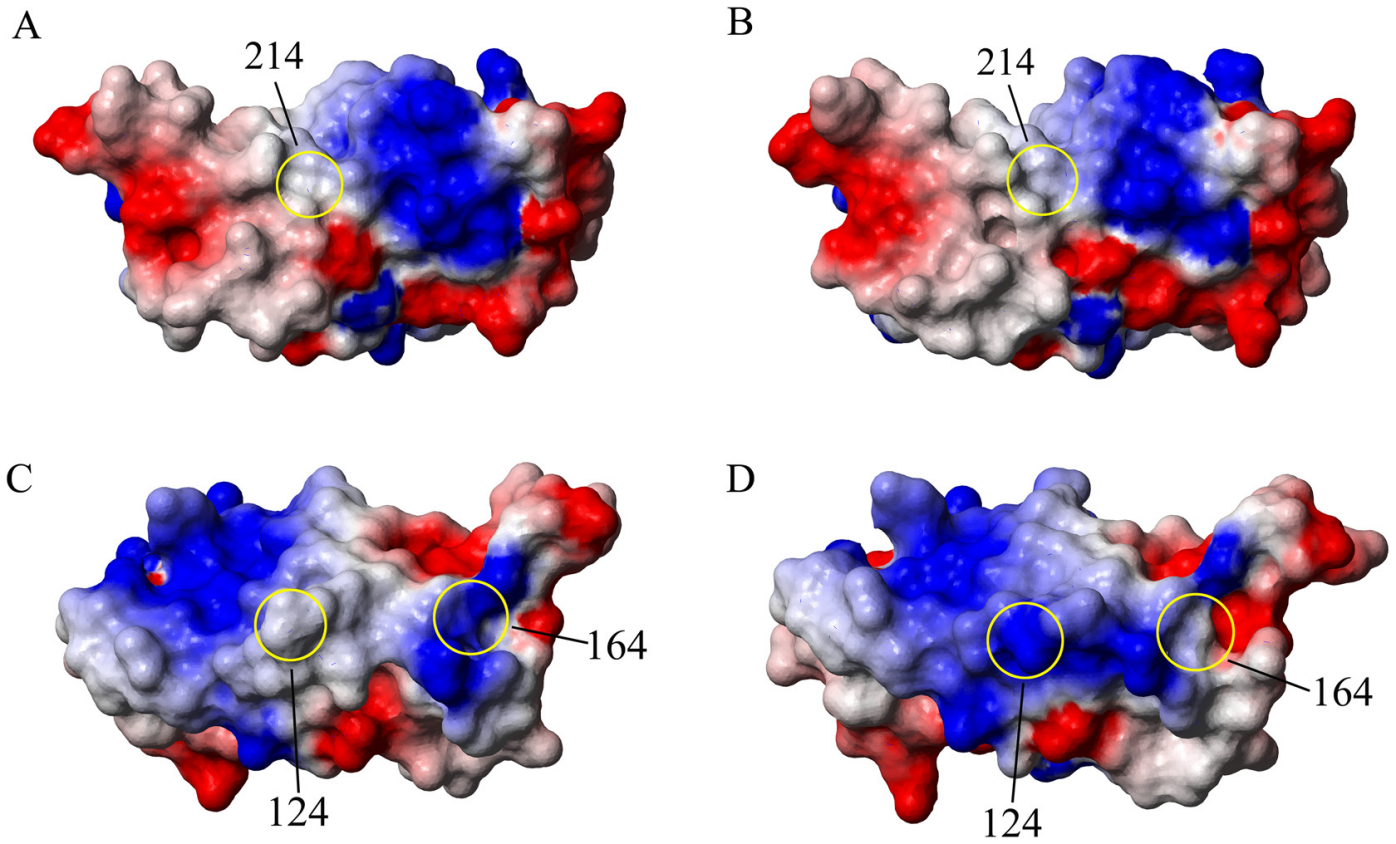
Distributions of surface electrostatic potential. Site 214 of (A) the wild-type and (B) the I214V mutant. Sites 124 and 164 of (C) the wild-type and (D) the I214V mutant. Positive, neutral and negative charges are colored blue, white and red, respectively. The surface diagrams are generated using MolMol.

We evaluated the effect of the I214V substitution on the surface charge distribution of RaPrP^C^(91–228). Both the mutant and the wild-type carry neutral charge at the right substituted site 214 (Fig. 2A, B). The I214V substitution does not change the electrostatic potential at site 214 due to the similar non-charged feature of the two amino acids. Unexpectedly, significant changes are observed at many other unsubstituted sites. For example, the wild-type displays a neutral charge distribution at site 124, while the I214V mutant carries positive charge in the same position (Fig. 2C, D). Site 164 also alters electrostatic potential from positive to neutral after the mutation (Fig. 2C, D). The I214V substitution results in a prominent shift of the surface charge distribution.

### Hydrogen bond

The hydrogen bond network usually makes a significant contribution to maintain secondary and tertiary structures of a protein. Stable hydrogen bonds are defined herein if they exist in at least 9 structures among the 15 lowest-energy structures. Totally, 41 hydrogen bonds are detected in the I214V mutant of RaPrP^C^(91–228), much less than the number 55 in the wild-type. In particular, only 4 hydrogen bonds participate in sustaining the tertiary structure of the mutant, compared with 8 in the wild-type (Table 2). As less hydrogen bonds are formed in the mutant, one could expect that the RaPrP^C^ molecule is somehow readily to experience the conformational conversion after the mutation.

**Table 2:** Hydrogen bonds maintaining the tertiary structures of RaPrP^C^(91–228) and the I214V mutant.

Protein	Hydrogen bonds
RaPrP^C^(91–228)	M128H^N^-Y162O, G130H^N^-V160O, I138H^N^-Y149O, R155HH12-D201OD1, R155HH22-Y148O, H176HD1-E210OE2, H186HD1-R155O, S221HG-D166OD1
I214V	G130H^N^-V160O, I138H^N^-Y149O, Y162H^N^- M128O, H186HD1-R155O

### Conformational stability

**Figure 3:**
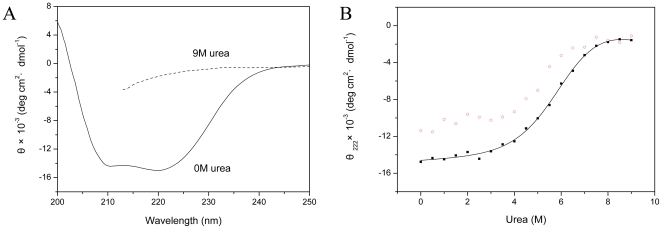
Urea-induced transitions of the I214V mutant of RaPrP^C^(121–228) characterized by CD spectroscopy. (A) The folded protein in the absence of urea (solid line) compared with the unfolded protein in the presence of 9 M urea (dashed line). (B) Mean residue ellipticity measured at 222 nm (θ_222_) in the presence of urea of different concentration. θ_222_ in denaturation is indicated by solid squares (▪), while in renaturation by open circles (○). The solid line presents the fitting curve on the basis of a two-state mechanism.

To access the conformational stability of the I214V mutant of RaPrP^C^(121–228), we performed urea-induced unfolding transition experiments using far-UV CD spectroscopy. The I214V mutant is rich in α-helix in the absence of urea (Fig. 3A), indicating a well-folded state. This mutant loses its secondary structure entirely under the condition of 9 M urea (Fig. 3A), implying a completely unfolded state. The mean residue ellipticity at 222 nm versus the urea concentration is shown in Fig. 3B, with a solid line showing the theoretical curve on the basis of a two-state mechanism. The denaturation was not reversible in our experimental condition (Fig. 3B), thus we determined the apparent thermodynamic parameters for the equilibrium unfolding of the I214V mutant (Table 3). 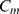 is the concentration of urea required to denature 50% of a protein, and 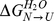 presents the apparent estimated free energy of unfolding extrapolated to zero concentration of denaturant. The measured values of 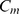 and 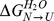 for the mutant are less than those for the wild-type (Table 3), indicating reduced conformational stability of RaPrP^C^ after the I214V substitution. The coefficient *m* is also different from that for the wild-type (Table 3).

**Table 3:** Apparent thermodynamic parameters for the equilibrium unfolding of RaPrP^C^(121–228) and the I214V mutant at 25°C.

Protein	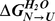 (kJ·mol^−1^)	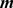 (kJ·mol^−1^·M^−1^)	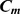 (M)
RaPrP^C^(121–228)	26.2±2.7	−3.88±0.49	6.49±0.05
I214V	16.1±1.8	−2.62±0.38	5.65±0.06

Note: 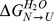 is an estimate of the free energy in the absence of denaturant, the parameter 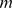 represents the cooperativity of the unfolding transition, and 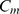 is the concentration of urea at the midpoint of unfolding. The determined parameters for the wild-type [30] are listed here to facilitate comparison.

### Relaxation rates R_1_, R_2_ and heteronuclear NOEs

**Figure 4:**
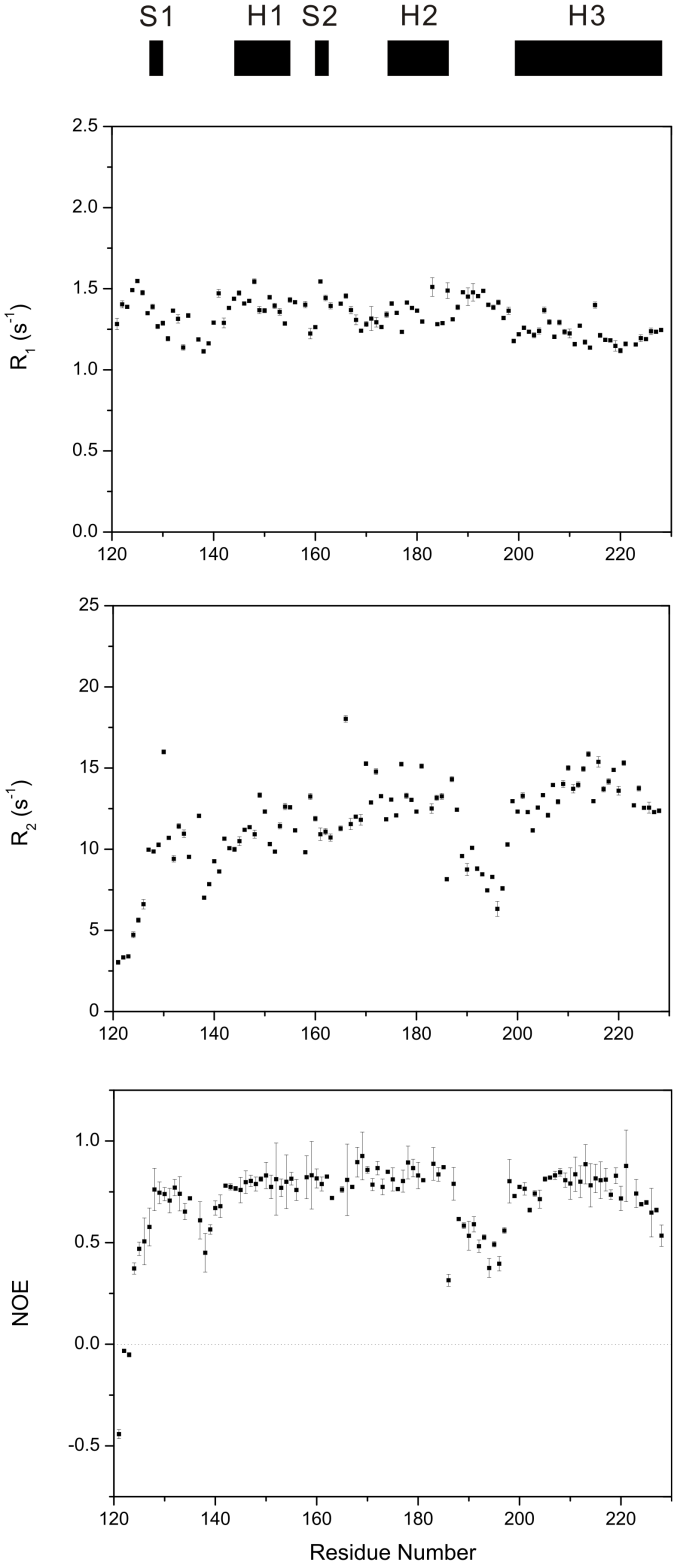
Relaxation rates R_1_, R_2_ and {^1^H}-^15^N heteronuclear NOEs of the I214V mutant of RaPrP^C^(121–228). Regular secondary structure elements are indicated on the top. The relaxation constants and the experimental errors were extracted by a single exponential curve fitting of the peak heights using Sparky (T. D. Goddard and D. G. Kneller, University of California, San Francisco).

To analyze the backbone dynamics of the I214V mutant of RaPrP^C^(121–228), we performed ^15^N relaxation measurements using NMR spectroscopy. As a whole, 103 assigned residues are used except 5 residues with unobservable resonance signals. We utilized the peak height for curve fitting so as to avoid the effect of partially overlapped resonance peaks. The relaxation rates R_1_, R_2_ and heteronuclear NOEs versus residue number are shown in Fig. 4. The R_1_ values do not change much with the sequence, mostly between 1.1 and 1.5 s^−1^. Different from the R_1_ distribution, the R_2_ values are relatively variable with residue number, ranging from 3.0 to 18.1 s^−1^ approximately. The residue D166 shows the largest R_2_ value over 18.1 s^−1^, and G130 also displays distinctly large R_2_ value as high as 16.0 s^−1^. Almost all residues exhibit positive NOE values except the first three residues. Typically, NOE values for the two antiparallel β-sheets and three α-helices are obviously higher than those for the loop fragments encompassing residues 121–127, 137–141 and 188–197. The secondary structure elements display NOE values larger than 0.7, indicative of more restricted dynamics in these regions.

### Reduced spectral density mapping

**Figure 5:**
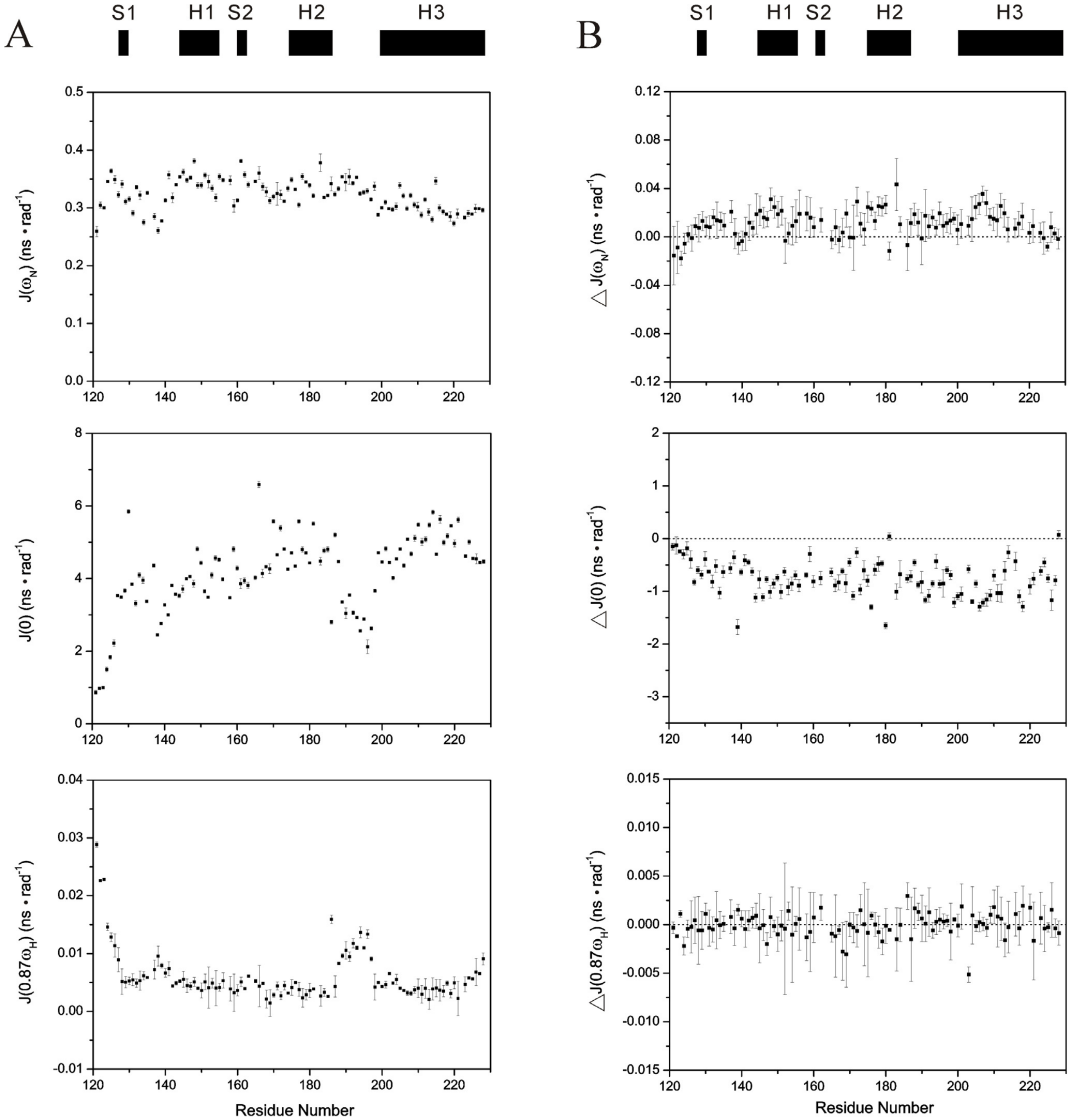
Reduced spectral density functions analysis. (A) Spectral densities of the I214V mutant of RaPrP^C^(121–228). (B) Differences of spectral densities between the wild-type and the I214V mutant. The difference is calculated as follows: Δ*J*(ω) = *J*(ω)_mutant_−*J*(ω)_wild-type_. Regular secondary structure elements are indicated on the top. The notebook provided by Spyracopoulos [62] was used to execute the calculation.

We adopted the spectral density function approach [31]–[33] to interpret the ^15^N relaxation data for the I214V mutant of RaPrP^C^(121–228). The calculated values of reduced spectral densities *J*(ω_N_), *J*(0) and *J*(0.87ω_H_) versus residue number are shown in Fig. 5A. The middle frequency spectral densities *J*(ω_N_) exhibit relatively invariable values ranging from 0.25 to 0.38 ns·rad^−1^, reflecting insensitivity to variations in backbone motion. Residues in helix 3 show a slight reduction in *J*(ω_N_) spectral densities compared to helices 1 and 2. It has been suggested that such a reduction reflects slightly anisotropic tumbling of PrP^C^ [34].

The low frequency spectral density *J*(0) is sensitive both to fast internal motions on the pico- to nanosecond (ps-ns) time scale and to slow motions (R_ex_) on the micro- to millisecond (µs-ms) time scale. Rapid internal motions tend to reduce the *J*(0) value, while slow internal motions usually lead to anomalously large *J*(0) values [35]. The plot of *J*(0) versus residue number shows the trend of R_2_ values change with the sequence since *J*(0) values are normally dominated by R_2_ values. In contrast to *J*(ω_N_), the spectral densities *J*(0) cover a wider range of values. Residues with lower *J*(0) values are mainly observed in loop regions 121–127, 138–141 and 189–197, implying sub-nanosecond flexibility of the N-H bond vector. Dissimilarly, loop 165–172 is not as flexible as the other three loop fragments. Residues in secondary structure elements have relatively higher *J*(0) values. However, the residue H186 in helix 2 displays a fairly small *J*(0) value, implying significant internal motion on the ps-ns time scale. D166 exhibits the largest *J*(0) value around 7.0 ns·rad^−1^, and G130 in β-sheet 1 also shows a remarkable *J*(0) value as large as 5.9 ns·rad^−1^, both indicative of slow µs-ms R_ex_ motions. Furthermore, a few residues in helices 2 and 3 are likely to undergo slow R_ex_ motions as well.

The high frequency spectral density *J*(0.87ω_H_) is sensitive only to fast internal motions on the sub-nanosecond time scale. Fast motions are reflected in relatively large values of *J*(0.87ω_H_). Aside from residues in loop regions 121–127, 138–141 and 189–197, the C-terminal end of helix 3 and the particular residue H186 show large *J*(0.87ω_H_) values, implying fast ps-ns motions. In addition, small *J*(0.87ω_H_) values and large *J*(0) values are observed in most residues in secondary structure elements, indicative of restricted motions on the fast ps-ns time scale.

The overall distributions of the reduced spectral densities *J*(ω_N_), *J*(0) and *J*(0.87ω_H_) for the I214V mutant are similar to those for the wild-type reported previously [30]. We compared the values of the spectral densities between the wild-type and the I214V mutant, and the differences versus residue number are shown in Fig. 5B. The most significant changes are observed in *J*(0). Almost all residues exhibit distinctly negative values in Δ*J*(0). Generally, the smaller the value of *J*(0), the greater the sub-nanosecond flexibility of N-H bond vectors [36]. Thus the I214V mutation leads to increased internal dynamics.

### Order parameter S^2^

**Figure 6:**
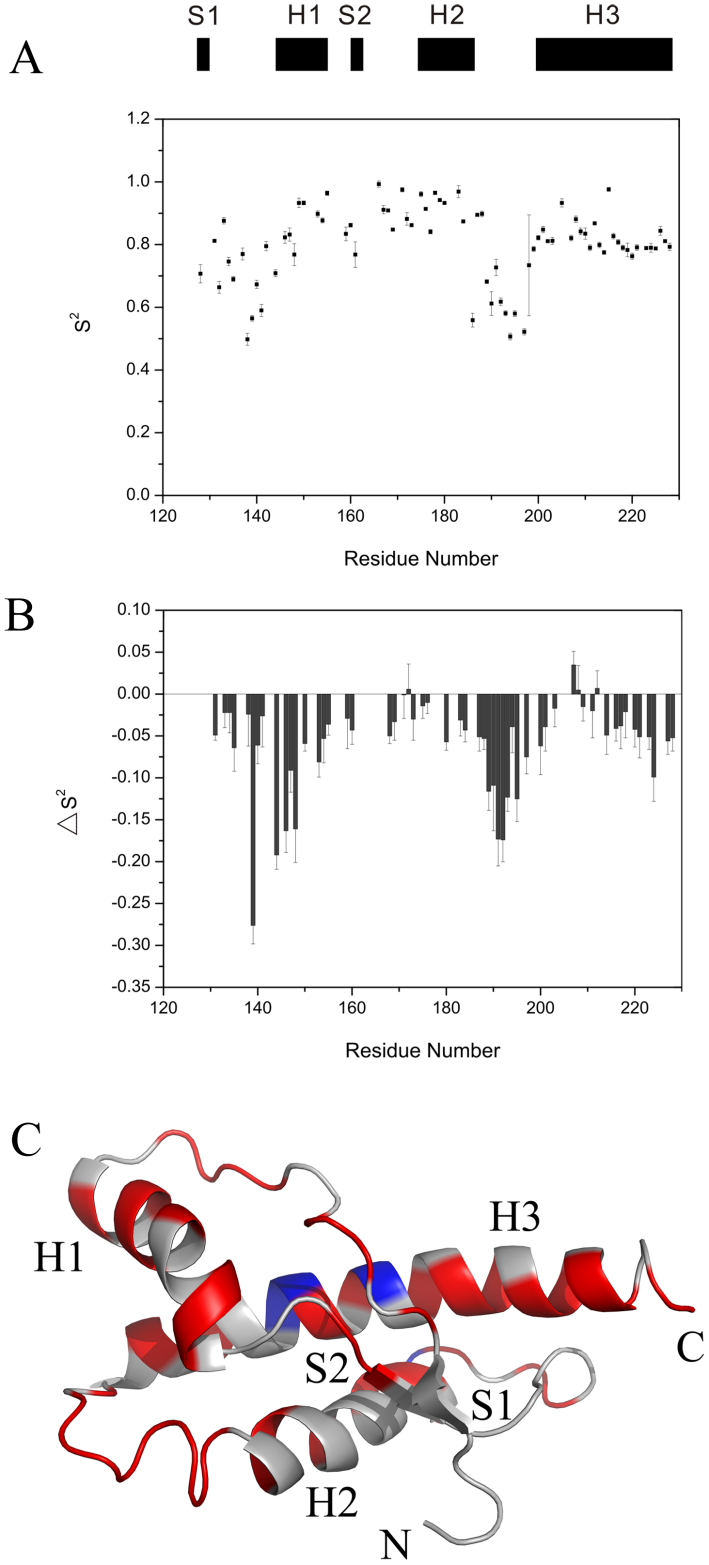
Modelfree analysis. (A) Order parameters S^2^ of the I214V mutant of RaPrP^C^(121–228). Regular secondary structure elements are indicated on the top. The program Fastmodelfree [63] was used to perform the calculation. Unavailable S^2^ values for a few residues are due to the absence of data or failure in the data fitting. (B) Differences in S^2^ values between the wild-type and the mutant. The difference is calculated according to the equation: ΔS^2^ = S^2^_mutant_−S^2^_wild-type_. The absence of ΔS^2^ values for residues result from unavailable S^2^ values for either the wild-type or the mutant. (C) ΔS^2^ values are mapped onto the tertiary structures of the I214V mutant: blue for ΔS^2^≥0, red for ΔS^2^<0, and grey for ΔS^2^ unavailable. This ribbon diagram is generated by PyMol (kindly provided by Prof. DeLano WL).

The modelfree formalism is usually used to analyze internal motions of a protein [37]–[39]. The D_∥_/D_⊥_ ratio of the rotational diffusion tensor for the I214V mutant of RaPrP^C^(121–228) was calculated to be 1.24±0.01 using the r2r1_diffusion program (kindly provided by Prof. A.G. Palmer 3rd), suggesting that the axially symmetric model is suitable for the data fitting. The calculated order parameter S^2^ versus residue number is plotted in Fig. 6A. This parameter describes the amplitude of the sub-nanosecond timescale motions (0≤S^2^≤1). Residues in loop 135–141 and 189–197 display the lowest S^2^ values, which imply that these regions are highly disordered. In contrast to these two loops, loop 165–172 show higher S^2^ values, indicating an ordered fragment. The S^2^ values for residues in secondary structure elements are relatively larger, which is consistent to the *J*(0) distribution. The mean values of S^2^ for helices 1, 2 and 3 are 0.88, 0.88 and 0.82, respectively. H186 in helix 2 shows a fairly small S^2^ value as low as 0.56, indicative of significant internal flexibility incompatible with the secondary structure property. We further compared the difference in S^2^ values between the wild-type and the mutant (Fig. 6B). Almost all residues show negative ΔS^2^ values, providing solid evidence that the I214V substitution significantly enhances the backbone flexibility of RaPrP^C^. The ΔS^2^ values are mapped onto the mean structure of the I214V mutant (Fig. 6C).

## Discussion

Single-point mutation may result in functional alteration owing to the global or local structural change in the protein. Several mPrP^C^ mutants exhibit local structural changes and show distinctly different behavior from the wild-type [29], [40]. Three single-residue mutants, including hPrP^C^(M166V), hPrP^C^(S170N) and hPrP^C^(R220K), show variations in the length and quality of definition of helix 3 [41]. In addition, loop 166–172, which is lack of backbone amide resonances in hPrP^C^ and mPrP^C^, is well-defined in both hPrP^C^ (S170N) and mPrP^C^ (V166A) mutants [16], [41].

In this present work, we have determined the solution structure of the I214V mutant of RaPrP^C^(91–228). The three-dimensional structure of the mutant is almost identical to that of the wild-type (Fig. 1), however, both the altered hydrogen bond network and the changed surface charge distribution demonstrate that the I214V substitution could lead to marked structural changes (Fig. 2 and Table 2). Significant change in surface-restricted charges has also been observed in hPrP^C^ (E200K) [42] and RaPrP^C^(S173N) mutants [30]. It is well known that electrostatic interaction could distinctly influence on both the binding specificity and affinity of a protein with substrates. Thus, the mutation-induced change of surface charge distribution is likely to affect the binding of PrP^C^ with many potential chaperones, including nucleic acids [43], [44], protein X [45], [46], sugars, lipids, etc., which would modulate the conformational conversion. Interestingly, both hPrP^C^(E200K) [42] and RaPrP^C^(S173N) mutants [30] exhibit distinct charge alterations at the right substituted sites, whereas the I214V mutant described herein remains the neutral charge at site 214 as wild-type RaPrP^C^ carries (Fig. 2). These observations suggest that the charge alteration at the substituted site is not a precondition for the global electrostatic potential shift. The change of surface charge distribution resulting from single-point mutation could be more significant than expected.

It has been demonstrated that both the tertiary structure and internal motions of a protein determine the protein function [47]. Therefore, evaluation of the differences in dynamics between wild-type PrP^C^ and its mutants is important to address the molecular mechanism of prion conversions. Both molecular dynamics simulations and NMR relaxation measurement experiments have suggested that backbone flexibility of PrP^C^ is associated with the conformational conversion [34], [48], [49], although some studies show that mutations do not lead to significant changes in backbone dynamics of proteins [40], [50]. Our results demonstrate that the I214V substitution leads to decreased *J*(0) and S^2^ values (Fig. 5 and 6), implicating an increase in backbone flexibility on the ps-ns time scale, similar to the S173N substitution [30]. Usually, fragments with high flexibility are indicative of low energy barrier to structural rearrangement. In addition, H186 in helix 2 displays a fairly small S^2^ value and a much lower *J*(0) value after the I214V mutation (Fig. 5 and 6), implying prominent internal motions which potentially make a significant contribution to destabilization of helix 2. Thus, it could be expected that the conformational change is somewhat easier to occur for the I214V mutant than for the wild-type. On the other hand, dynamics could modulate binding energy to maintain the moderate affinity required for biological function [51], [52]. The increased dynamics might enhance or inhibit the binding ability of PrP^C^ with other chaperones, which would exert profound influence on prion conversions.

We have assessed the change of thermodynamic stability of RaPrP^C^ after the I214V substitution. The coefficient *m* is implicated to be approximately proportional to the difference in solvent-accessible surface areas between the folded and unfolded states. Hydrophobic interactions stabilizing the protein native state can be interpreted, to a large extent, in terms of the change in accessible surface area upon protein folding [53]–[56]. The distinctly changed *m* value is indicative of differences in intramolecular hydrophobic interactions between the wild-type and the mutant (Table 3). Furthermore, similar to the S173 substitution [30], the I214V substitution leads to marked decreases in both 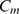 and 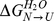 values during urea-induced transitions (Fig. 3 and Table 3), implying that the I214V mutant adopts a less stable conformation compared with the wild-type. Our experimental observations are supported by both molecular dynamics simulations reported recently [57] and the altered hydrogen bond network described above (Table 2). It has also been shown that internal dynamics especially the ps-ns motions has an intimate correlation with thermodynamics [58], [59]. The increased backbone flexibility, indicated by the decreased *J*(0) and S^2^ values (Fig. 5 and 6), implying the conformational instability of the I214V mutant. Therefore, one could expect that PrP^C^ with low structural stability would be inclined to undergo conformational conversion.

In conclusion, the I214V substitution results in distinct structural changes for RaPrP^C^. Both the unique surface charge distribution and the intrinsic backbone are likely to make significant contributions to species barriers for the transmission of TSEs. Our results provide valuable hints for understanding the inability of RaPrP^C^ to form PrP^Sc^, and shed light on the detailed molecular mechanism of the conformation conversion for prion proteins.

## Materials and Methods

The I214V mutants of RaPrP^C^(91–228) and RaPrP^C^(121–228) were constructed using site-directed mutagenesis PCR. Plasmid construction, protein expression and purification, NMR spectroscopy, structure calculation, ^15^N relaxation measurements, dynamics analysis and CD experiments were conducted as described previously [30], [60]. Nearly complete backbone and side-chain resonance assignments for the I214V mutant of RaPrP^C^(91–228) have been obtained. The chemical shift data are available at the Biological Magnetic Resonance Data Bank with accession number 16616. The atomic coordinates have been deposited into the Protein Data Bank with PDB ID 2JOM.
